# Supplementary material for: Influences of Excluded Volume of Molecules on Signaling Processes on the Biomembrane
Source: PLoS One. 2013 May 2;8(5):e62218. doi: 10.1371/journal.pone.0062218 (PMC3642174; doi:10.1371/journal.pone.0062218)
Supplement: Text S1 — Mean-field approximation. (PDF) [file pone.0062218.s001.pdf]

Supporting Information Text S1:

Influences of Excluded Volume of Molecules on Signaling Processes on the Biomembrane

Masashi Fujii*, Hiraku Nishimori, Akinori Awazu

Mean-field approximation

We analyzed the present model by mean-field approximation based on the mass action law. For simplicity, we showed only the results of the system without crowders because the results were qualitatively the same as that for the system with crowders. The temporal evolution of the occupancy of each molecular species, $[R]$, $[R^*]$, $[S^*]$, $[S]$, and $[T]$, is obtained by

$$\frac{d[R]}{dt} = -k_R[R] + k_{R^*}[R^*] \tag{S1}$$
$$\frac{d[R^*]}{dt} = k_R[R] - k_{R^*}[R^*] \tag{S2}$$
$$\frac{d[S]}{dt} = -k_S[R^*][S] + k_{S^*}[S^*] \tag{S3}$$
$$\frac{d[S^*]}{dt} = k_S[R^*][S] - k_{S^*}[S^*] \tag{S4}$$
$$\frac{d[T]}{dt} = (1-\rho)P_{in} - k_T[T][S^*] - P_{out}[T] \tag{S5}$$
$$\rho = [S_{tot}] + [R_{tot}] + [T]. \tag{S6}$$

From these equations, we estimated the steady-state signal flow, defined as the frequency of activation for unbinding of the target protein, given by

$$J = k_T[T][S^*] = \frac{aP_{in}(1-[R_{tot}]-[S_{tot}])}{P_{in}+P_{out}+a},$$
$$a = \frac{k_R k_S k_T[R_{tot}][S_{tot}]}{k_R k_{S^*} + k_{R^*}k_{S^*} + k_R k_S[R_{tot}]}. \tag{S7}$$

This equation clearly exhibits a Michaelis-Menten-type equation in terms of P_{in}, subsequently, J monotonically increases with P_{in} independent of $[S_{tot}]$ and P_{out}.
