# Supplementary material for: Influences of Excluded Volume of Molecules on Signaling Processes on the Biomembrane
Source: PLoS One. 2013 May 2;8(5):e62218. doi: 10.1371/journal.pone.0062218 (PMC3642174; doi:10.1371/journal.pone.0062218)
Supplement: Text S2 — Spatial-temporal scale and volume fraction of the present model. (PDF) [file pone.0062218.s002.pdf]

Supporting Information Text S2:

Influences of Excluded Volume of Molecules on Signaling Processes on the Biomembrane

Masashi Fujii*, Hiraku Nishimori, Akinori Awazu

Spatial-temporal scale and volume fraction of the present model

We considered the relationship between the spatial-temporal scales of the present model and those of experiments. First, we considered the unit length and the unit time in the present model.

If we regard the present model as the GPCR signal transduction system, its spatial scales are estimated by considering the size of each cell to be of the same order as its molecular size, i.e., nm. The diffusion rate of molecules was estimated as $\sim 10^{-1}$–$10^{-2}\mu\text{m}^2/\text{s} = 10^{-1}$–$10^{-2}\text{nm}^2/\mu\text{s}$ for the GPCR without the cytoskeleton [1]. Hence, the time interval in which a molecule moves a single molecule length is estimated as $\sim$ 10–100μs. This time interval is the unit time step in the model. We also assumed that the 0.01–1 reactions occur for each molecule at each time step. This assumption yields the characteristic time for reactions as 0.1–10ms, which is consistent with the time scale of the conformational change of typical proteins.

Next, we considered the relationship between occupancy in the present argument and the volume fraction of molecules. In general, the detailed relationship between the two is nontrivial because it depends on the detailed properties of the considered system, such as the sizes and shapes of the molecules and environmental components. However, if all molecules are considered spheres with almost the same radii, the volume fraction is roughly estimated from the occupancy, as in the following.

In the present model, each cell could contain only one molecule. This implies that the size of each cell was larger than that of each molecule, but had to be small enough for the distance between 2 molecules in neighboring cells to be always smaller than the molecular diameter, to avoid the possibility of the invasion of a molecule between 2 neighboring molecules. Then, the length of the diagonal of each cell, d, and the radius of the molecules, r, always had to satisfy the equation

$$\sqrt{\left(\frac{\sqrt{3}d}{2}-r\right)^2+\left(\frac{d}{4}-\frac{r}{\sqrt{3}}\right)^2}-r \leq r. \tag{S8}$$

According to such d and r, the ratio of the volume fraction to the occupancy is obtained by

$$\frac{[\text{Volume fraction}]}{[\text{Occupancy}]} = \frac{[\text{area of one molecule}]}{[\text{area of one cell}]} = \frac{8\pi r^2}{3\sqrt{3}d^2}. \tag{S9}$$

For example, we assumed that the length of the diagonal of each hexagonal cell was $\sim 1.7\times$ [molecular diameter], which is close to the largest value to satisfy Equation S8. Then, the volume fraction of the densest condition with occupancy $= 1$ in the 2-dimensional cell-based model was estimated as $\sim 41\%$, which is a slightly large but possible value in experimental situations [2].

SI Reference

1. Suzuki K, Ritchie K, Kajikawa E, Fujiwara T, Kusumi A (2005) *Rapid hop diffusion of a G-protein-coupled receptor in the plasma membrane as revealed by single-molecule techniques.* Biophys J **88**: 3659–3680.

2. Fulton AB (1982) *How crowded is the cytoplasm?* Cell **30**: 345–347.
