# Supplementary material for: Influences of Excluded Volume of Molecules on Signaling Processes on the Biomembrane
Source: PLoS One. 2013 May 2;8(5):e62218. doi: 10.1371/journal.pone.0062218 (PMC3642174; doi:10.1371/journal.pone.0062218)
Supplement: Text S3 — Details of quantification. (PDF) [file pone.0062218.s003.pdf]

Supporting Information Text S3:

Influences of Excluded Volume of Molecules on Signaling Processes on the Biomembrane

Masashi Fujii*, Hiraku Nishimori, Akinori Awazu

Details of quantification

In the simulation, the local occupancy of molecule X, $\rho_X(r)$, was calculated as follows. First, we defined the distances from the receptor to the molecules of the cell as the minimum number of steps to move from the receptor to the molecules. Second, we used $N_X(r)$ and $N(r)$ as the number of molecule X and cells at a distance r from the receptor to the molecules, respetively. Then,

$$\rho_S(r) = \frac{N_X(r)}{N(r)} \tag{S10}$$
